# Dementia epidemiology in Hungary based on data from neurological and psychiatric specialty services

**DOI:** 10.1038/s41598-021-89179-3

**Published:** 2021-05-14

**Authors:** Nóra Balázs, András Ajtay, Ferenc Oberfrank, Dániel Bereczki, Tibor Kovács

**Affiliations:** 1 Department of Neurology, Semmelweis University, Budapest Balassa utca 6., Budapest, 1083 Hungary; 2 MTA-SE Neuroepidemiological Research Group ELKH, Budapest Balassa utca 6., Budapest, 1083 Hungary; 3 Institute of Experimental Medicine, Budapest Szigony utca 43., Budapest, 1083 Hungary

**Keywords:** Medical research, Neurology

## Abstract

Hungary has a single-payer health insurance system covering 10 million inhabitants. All medical reports of the in- and outpatient specialist services were collected in the NEUROHUN database. We used ICD-10 codes of Alzheimer’s disease (AD), vascular dementia (VaD), miscellaneous dementia group and mild cognitive impairment (MCI) for the inclusion of the patients. Incidence, prevalence and survival of different dementias and MCI were calculated and analyzed depending on the diagnoses given by neurological or psychiatric services or both. Between 2011 and 2016, the mean crude incidence of all dementias was 242/100,000/year, whereas the age standardized incidence was 287/100,000/year. Crude and age standardized mean prevalence rates were 570/100,000 and 649/100,000, respectively. There were significantly more VaD diagnoses than AD, the VaD:AD ratio was 2.54:1, being the highest in patients with psychiatric diagnoses only (4.85:1) and the lowest in patients with only neurological diagnoses (1.32:1). The median survival after the first diagnosis was 3.01 years regarding all dementia cases. Compared to international estimates, the prevalence of dementia and MCI is considerably lower in Hungary and the VaD:AD ratio is reversed.

## Introduction

Dementia, as a syndrome, is comprised of acquired cognitive and behavioral symptoms which are sufficiently severe enough to cause impairment in everyday and/or occupational activity of the patient, while the diagnosis of mild cognitive impairment (MCI) is used to describe symptoms that are measurable by cognitive testing but do not interfere with functional abilities^1^. The most common causes of dementia in older adults (> 65 years) are Alzheimer’s disease (AD), vascular (VaD) and Lewy body dementias^2^.

In aging societies, dementia is receiving increased attention due to its significant healthcare, societal and economic burden. Approximately 45–50 million people lived with dementia worldwide in 2015, and this number is expected to increase to 130 million by 2050. In addition to its adverse effects on patients' and caregivers’ quality of life and life expectancy, huge economic burden for the society is imposed by the increase in the prevalence of dementia: its cost was estimated to be $818 billion in 2015^3,4^.

Analyzing and comparing epidemiological data from countries with different geographical and economic characteristics could be a useful opportunity to gain a better understanding of dementia as a multifactorial syndrome. Overall, population-based surveys should be conducted and regularly repeated in all countries to monitor changes in trends^5^.

Hungary is a country with 10 million inhabitants and the whole population is covered by a single-payer state health insurance system. Inpatient and outpatient care are documented in a unified system at a nationwide level enabling comprehensive data collection.

Our aim was to estimate the prevalence of MCI and dementia (with its subtypes) in Hungary using data from the health insurance database and to compare the Hungarian data with the international ones.

## Methods

Our study was performed using the NEUROHUN 2004–2017 database, which was created from medical and medication prescription reports within the framework of the Hungarian National Brain Research Program^6^. Appearances in all in- and outpatient departments (except family medicine (FM)) in Hungary in the indicated period are documented in the database. In our study, data from 2011 to 2016 were analyzed. The original patient identifier codes were anonymized and encrypted identifiers were used.

All personal data protection regulations were followed. The study was approved by the Ethics Committee of Semmelweis University, Budapest, Hungary (Approval No: SE TUKEB 88/2015).

### Selection of patients

**Figure 1:**
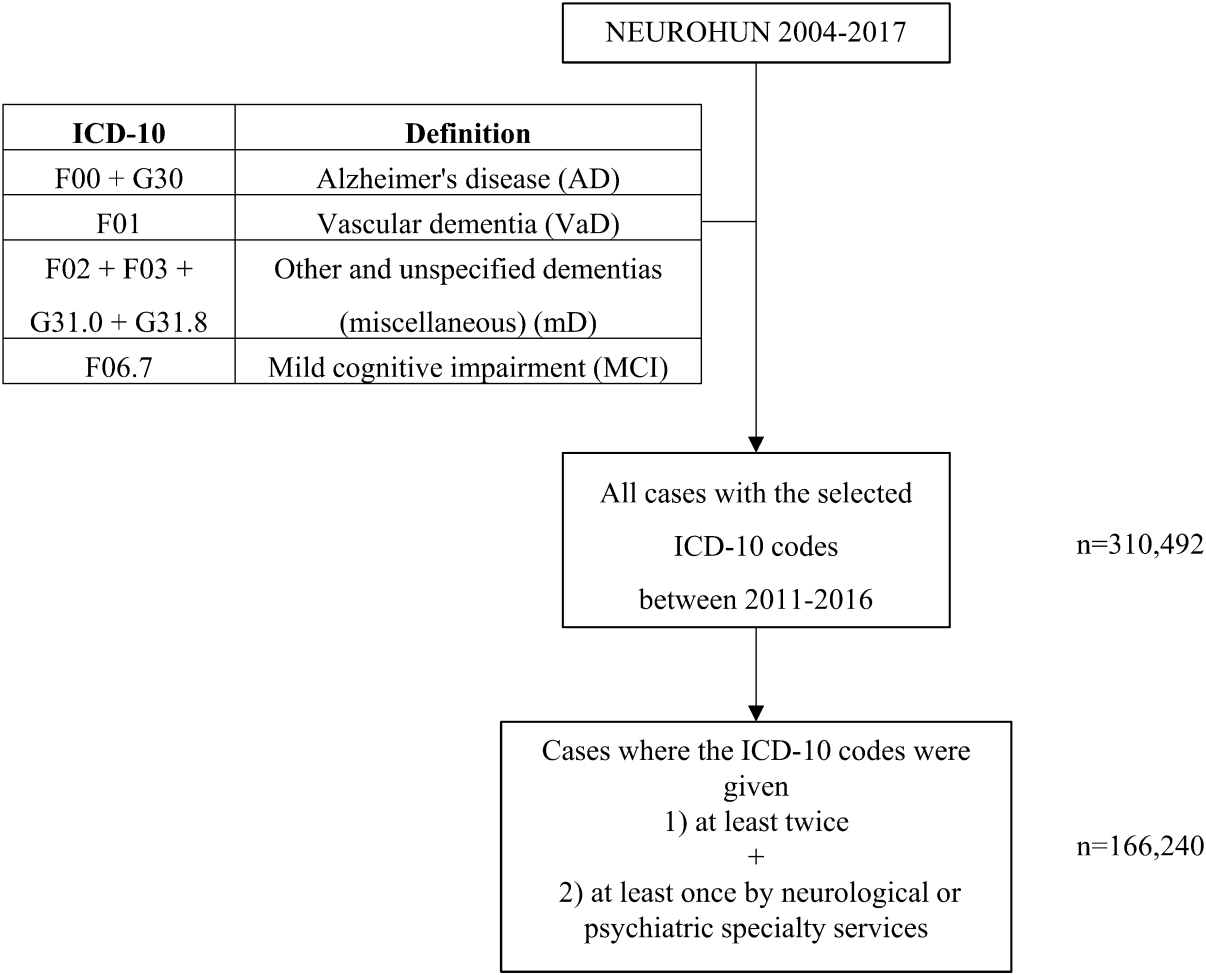
Flowchart of patient selection and the classification of subtypes of dementia (with their abbreviations) used in our study with their corresponding ICD-10 codes.

First, all patients with a diagnosis of dementia and MCI were collected. Cases were categorized based on diagnoses according to the International Classification of Diseases (ICD-10). Although criticism is raised in the literature regarding the use of ICD-10 codes^7^, these are the exclusively used ones in the health insurance database. Only those ICD codes were selected which were given by medical specialty services; diagnostic and non-medical services (e.g. physiotherapy, psychology) were excluded. For the analysis, we used data of those patients only who had been assigned with dementia or MCI ICD codes at least twice and at least one of the ICD codes was given by neurological or psychiatric specialty services (Fig. 1).

### Validation of the database

Validation of the clinical diagnosis criteria of dementias on a smaller subsample was performed. We checked patients who had records with the defined ICD codes in the local integrated hospital healthcare information technology system (MedSol, T-System, Hungary) of Semmelweis University, Budapest, in a selected period (October 2013) and compared them to the records in the NEUROHUN database. To match patients across the databases, we used the year of birth, postal code of the residence, the gender, the admission and the discharge date, together with the institutional code of the medical service provider (Department of Neurology, Semmelweis University, Budapest, Hungary).

First, we collected all records provided by our department in the selected period of time in the NEUROHUN database, then we checked whether these patients could be found in MedSol.

Second, we identified all patients at our department in MedSol who were treated with these ICD-10 codes either in the inpatient or the outpatient setting. Then, we checked these patients whether they did or did not appear in the NEUROHUN database.

Finally, for further clarification, we reviewed the medical records of these patients to ensure that the clinical findings support the diagnosis of dementia.

### Statistical analysis

We calculated crude and age-standardized^8^ incidence and prevalence rates of dementias for each year for the period of 2011–2016. The newly diagnosed cases were analyzed by gender and age groups. For the analysis we used the date of first application of the diagnoses (without preceding diagnosis in the NEUROHUN database).

AD and VaD groups were analyzed separately. ICD-10 codes of G31.0 (corresponding to frontotemporal dementia with clinically heterogeneous diseases) and G31.8 (including diseases other than Lewy body dementias too) were included in the group of miscellaneous dementias (mD).

Population data were used from the database of the Hungarian Central Statistical Office based on the census in 2011^9^.

Microsoft Excel 2016 (Microsoft Corporation, Redmond, Washington, USA) (descriptive statistics), TIBCO Statistica version 13 (TIBCO Software Inc, Palo Alto, California, USA) (one- and two-way ANOVAs) and GraphPad Prism 8 (GraphPad Software Inc., San Diego, California, USA) (Kaplan–Meier survival curves, log rank tests) were used for analyses.

## Results

### Validation of the database

In October 2013, 124 patients were registered in the MedSol system, of which 122 cases were found in the NEUROHUN database. The two missing patients from the NEUROHUN had a reporting error from the hospital and as a result, were not financed, so they did not appear in the NEUROHUN database. From the other direction, we were able to identify all 122 patients from NEUROHUN in MedSol.

### Estimating the number of patients with dementia and MCI

During the examined six-year period, more than 1,956,000 (689,000 neurological, 1,087,000 psychiatric) appearances of 144,407 patients in the Hungarian health care system were associated with any type of dementia diagnosis and 467,063 (148,773 neurological and 318,290 psychiatric) appearances of 21,833 patients with MCI alone. At least one computed tomography (CT) scan of the head was performed in 70.3% (n = 101,559) of the patients, while only 12.0% (n = 17,339) had a magnetic resonance imaging (MRI) of the head. The proportion of patients without head CT or MRI was 26.2% (n = 37,838).

The number of new patients diagnosed with dementia during the observed period and the calculated incidence and prevalence results are summarized in Table 1.

**Table 1:** Summary of number, prevalence and incidence data of patients and dementia types between 2011 and 2016.

Year	Number of new patients^a^	AD^a^	VaD^a^	mD^a^	Number of dementia diagnoses^a^	All dementias^b^				AD alone^b^				MCI^b^			
						Preval.		Incid.		Preval.		Incid.		Preval.		Incid.	
						St	Cr	St	Cr	St	Cr	St	Cr	St	Cr	St	Cr
2011	32,115	4859	16,723	17,026	38,608	314	270	379	323	17	13	17	15	84	67	87	69
2012	27,168	5377	15,389	16,062	36,828	519	450	322	273	27	24	16	14	149	125	70	62
2013	25,022	5609	14,916	15,993	36,518	665	580	299	252	38	33	17	14	213	183	70	63
2014	23,102	6010	14,077	15,659	35,746	767	673	277	232	50	44	18	15	275	235	70	58
2015	20,800	6117	13,159	14,898	34,174	819	724	250	209	62	55	20	17	332	285	66	58
2016	16,200	5612	11,094	12,432	28,138	810	722	195	163	75	66	20	17	376	324	54	47
Total/mean^c^	**144,407**	**33,584**	**85,358**	**92,070**	**211,012**	**649**	**570**	**287**	**242**	**45**	**39**	**18**	**15**	**238**	**203**	**70**	**59**

^a^Number of new patients and types of dementia. The date of the first diagnosis of any dementia type given to a patient was used for new patients; patients having more than one diagnosis were not defined as new patients when their diagnosis changed.

^b^Incidence (new patients per 100,000 inhabitants/year) and prevalence (number of patients per 100,000 inhabitants) of all types of dementia, AD alone (without the mixed cases) and MCI in Hungary between 2011 and 2016. Age standardization was performed using the 2013 European standard population^8^. *Incid.* incidence, *Preval.* prevalence, *Cr* crude, *St* standardized.

^c^The total numbers in case of ^a^columns and the mean values of incidences and prevalences.

A notable number of patients received multiple dementia diagnoses. Of the 33,584 AD patients, only 27% (n = 9165) were diagnosed with AD alone (with or without MCI diagnosis), 73% of the cases were associated with other subtypes of dementia. AD diagnosis was more often associated with mD (n = 17,451) than with VaD (n = 15,140).

### Association of MCI

**Figure 2:**
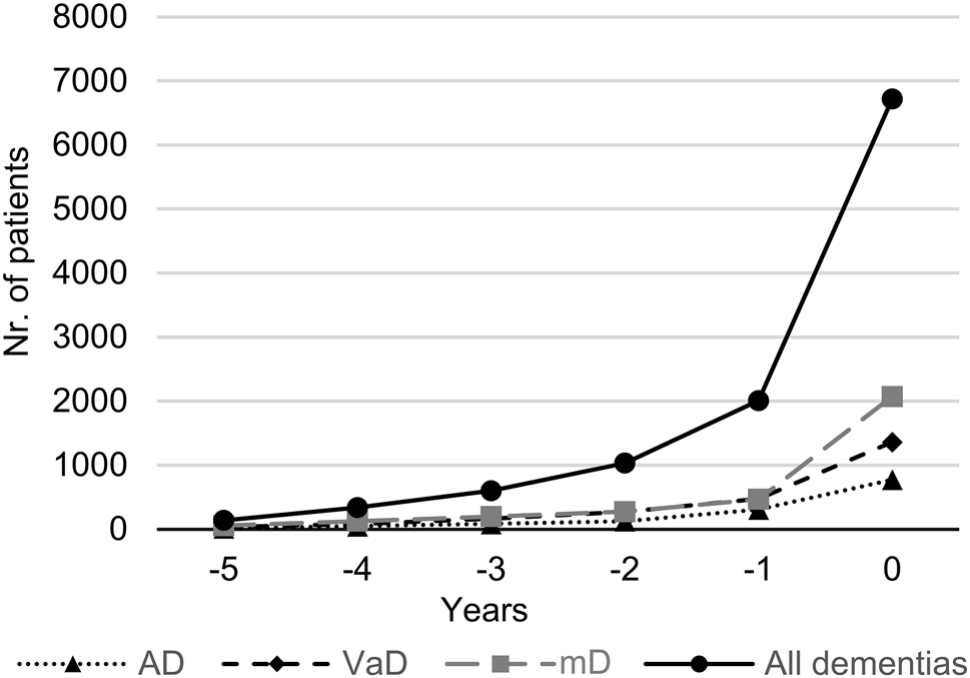
The relationship between types of dementia and MCI. MCI assigned to patients with AD, VaD, mD and all dementia diagnoses. In case of zero, the patient received MCI and another diagnosis in the same year. The date of the first diagnosis given to a patient was used for the analysis. *Nr.* Number.

The association of MCI with different types of dementia is shown in Fig. 2. During the 6-year period, the number of patients without a diagnosis of dementia but receiving MCI was 21,833. Of the 9165 patients diagnosed with AD alone, 16.48% (n = 1510) had the diagnosis of MCI at some point during the course of the disease, but MCI was diagnosed only in 14.81% (n = 1357) preceding the diagnosis of AD. The same data for VaD were 8.05% (n = 2913) and 6.61% (n = 2392), for mD 10.05% (n = 4081) and 7.88% (n = 3198), while for all dementias 9.34% (n = 13,487) and 7.51% (n = 10,838), respectively.

### Analysis of dementia subtypes

**Figure 3:**
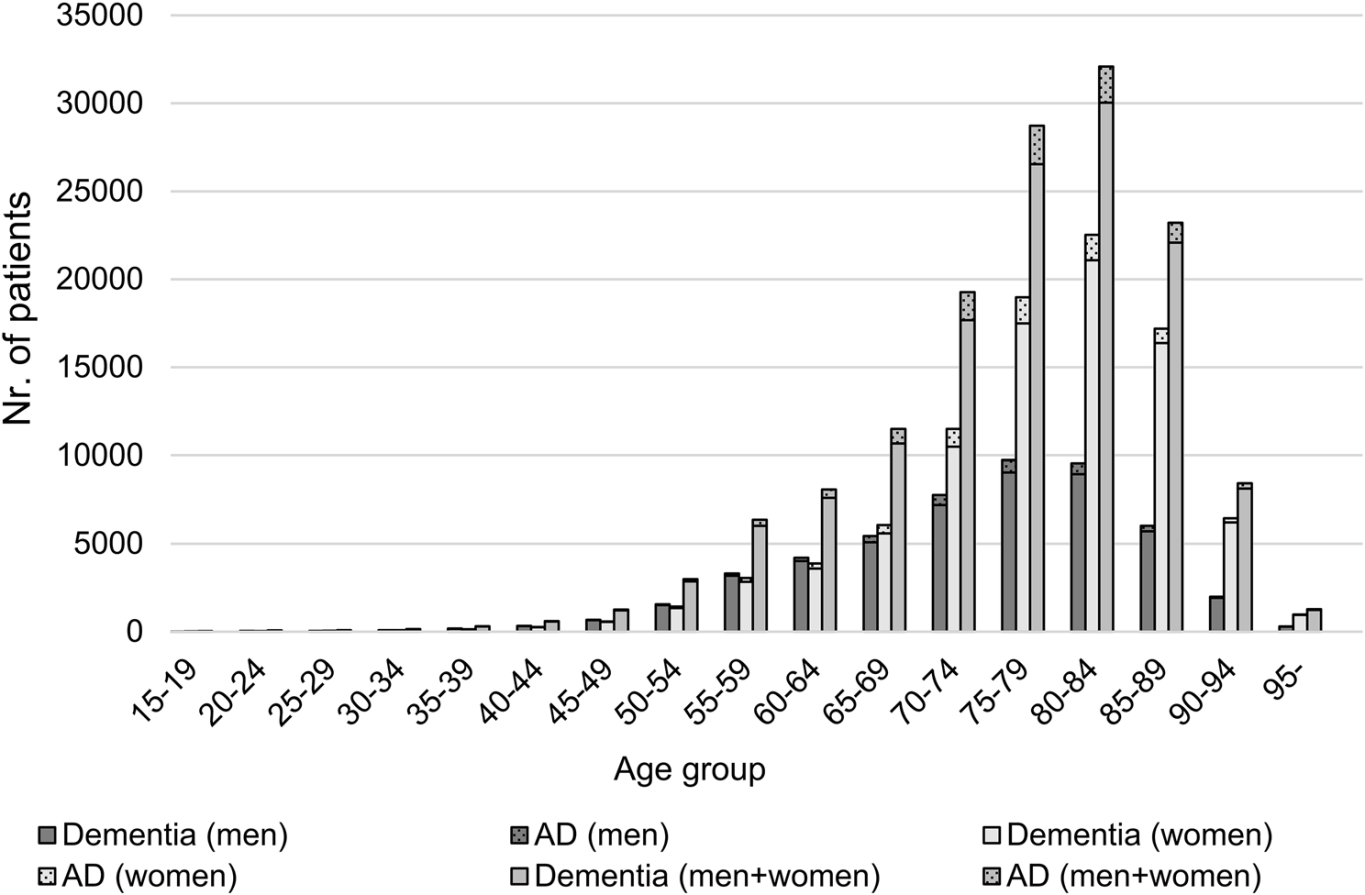
Cases of dementias and AD between 2011 and 2016 by age groups. The bars indicate the total dementia, of which AD is highlighted with dotting.

When examined by age groups, more men were diagnosed with dementia between the ages of 35 and 65, while the proportion of women under the age of 35 and over 65 was higher. In the total sample, more women were diagnosed with AD than men. With advancing age, the proportion of people diagnosed with AD is increased in both genders, peaking in the 80–84 age group, followed by a decrease in the incidence from the age of 85, with male predominance (Fig. 3).

**Figure 4:**
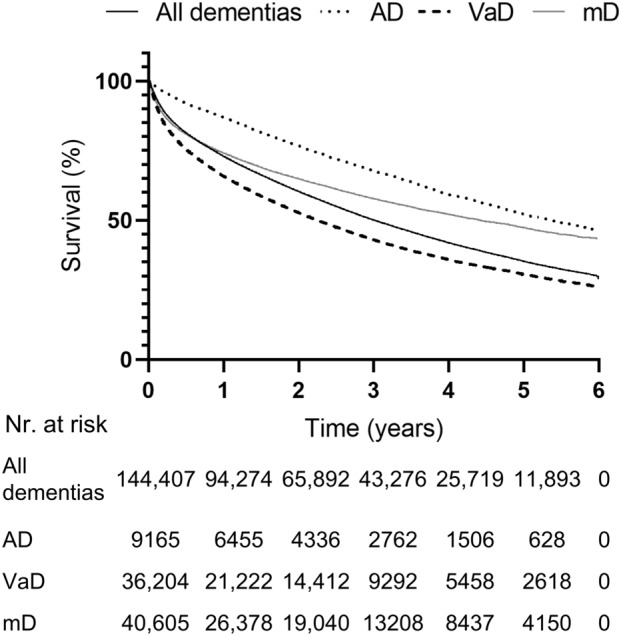
Survival from the diagnosis of dementia subtypes plotted on a Kaplan–Meier curve, logrank test p < 0.0001.

The mean age is between 70 and 80 years for all forms of dementia, with a statistically significant difference by the types of dementia and gender except AD (men) vs. VaD (men) as well as AD (men) and VaD (men) vs mD (women) (two-way ANOVA, post hoc Tukey HSD p < 0.0001, for the matrix, see Supplementary information 1). In all dementia types, the mean age was lower in men. Case fatality was the highest in VaD and in all types the rate of death was higher in men. The median survival after the first diagnosis in all dementia cases was 3.01 years. There was a significant difference between the types: survival was the longest with AD and the shortest with VaD (Table 2 and Fig. 4).

**Table 2:** Main features of dementia types.

Gender	All dementias	AD	VaD	mD
**Age (mean ± standard deviation)**				
Overall	76.48 ± 10.62	76.01 ± 9.12	77.80 ± 10.17	73.28 ± 12.66
Men	73.82 ± 11.21	74.88 ± 9.17	75.02 ± 10.85	70.16 ± 12.79
Women	77.94 ± 9.99	76.56 ± 9.04	79.22 ± 9.51	75.24 ± 12.18
**Observed deaths (number of cases, %)**				
Overall	73,349 (50.79%)	2662 (29.05%)	20,529 (56.70%)	16,985 (41.83%)
Men	27,452 (53.62%)	934 (30.98%)	7211 (59.16%)	6795 (43.36%)
Women	45,897 (49.24%)	1728 (28.10%)	13,318 (36.79%)	10,190 (40.86%)
**Median (95% Confidence interval) years of survival after diagnosis**				
Overall	3.01 (2.98–3.04)	5.37 (5.07–5.75)	2.25 (2.20–2.30)	4.42 (4.30–4.54)
Men	2.68 (2.64–2.74)	4.82 (4.42–5.61)	1.99 (1.90–2.07)	4.20 (4.00–4.39)
Women	3.20 (3.16–3.24)	5.43 (5.18–5.92)	2.39 (2.33–2.46)	4.57 (4.40–4.73)

There was statistically significant difference in mean age by types of dementia and gender except AD (men) vs. VaD (men) as well as AD (men) and VaD (men) vs mD (women) (two-way ANOVA, post hoc Tukey HSD p < 0.0001). In the case of observed death, we compared the number of deaths over six years to the total number of patients. The median survivals were significantly different (log rank test p < 0.0001).

Dementia diagnoses were given by psychiatric specialty alone in 45.1% (n = 65,118) of the cases, while in 20.6% (n = 29,720) and in 34.3% (n = 49,569) by neurological and both specialties, respectively. There was a significant difference in the mean age (one-way ANOVA, p < 0.00002) and median survival after the diagnosis (logrank test, p < 0.0001) between the three groups: the highest mean age and the shortest survival was observed in patients with diagnoses given by psychiatric specialties only. The survival was the longest in patients with neurological diagnosis.

The VaD:AD ratio was the highest in patients with psychiatric diagnoses only (4.85:1) and was the lowest in patients with only neurological diagnoses (1.32:1). Two-thirds of the patients without neuroimaging were in the group diagnosed by psychiatric specialty services alone (39.0% (n = 25,393) of the patients diagnosed by psychiatric specialty services only). The lowest rate of patients (13.1%, n = 6505) without neuroimaging was seen in patients diagnosed by both specialties (Table 3).

**Table 3:** Comparison of the neurological and psychiatric diagnoses.

ICD-10 code given by	Neurologist	Psychiatrist	Both
Nr. of patients	29,720	65,118	49,569
Nr. of AD diagnoses	8980	8873	15,731
Nr. of VaD diagnoses	11,869	43,024	30,465
VaD:AD ratio	1.32:1	4.85:1	1.93:1
Age (mean ± standard deviation)	74.39 ± 11.61*	77.61 ± 10.61*	76.27 ± 9.65*
Median (95% Confidence interval) years of survival after diagnosis	5.23 (5.11–5.41)*	2.25 (2.21–2.29)*	3.14 (3.08–3.19)*
Nr. of patients without head imaging	5940 (20.0%)	25,393 (39.0%)	6505 (13.1%)

Patients were categorized according to which specialty gave the dementia diagnosis. The three groups are patient who were diagnosed only by neurological or psychiatric specialty or got diagnosis from both specialties. AD and VaD categories include the mixed pathologies as well. Differences between the mean age were significant (one-way ANOVA, *p < 0.00002), the same was observed between the survival after the dementia diagnosis (logrank test, *p < 0.0001).

*Nr.* Number.

## Discussion

AD and VaD are the two most common types of dementia. Based on clinical diagnostic criteria the ratio of VaD to AD is approximately 1:3 in Europe and in the United States, while in the developing countries it is 1:2^10–12^. With advancing age, the incidence of degenerative and vascular diseases is increasing, together with the increase of vascular risk factors involved in the pathogenesis of AD; as a result of this, the frequency of dementias with mixed etiologies is increasing^13,14^.

According to the meta-analysis of Prince^5^, there are few epidemiological studies from the Central and Eastern European regions. However, data from the few available studies^15,16^ suggest that prevalence in these regions are similar to the Western European countries in terms of cognitive impairment and dementia. Based on these, the standardized prevalence among over 60 years of age is between 7.1 and 7.3% (meaning about 160–170,000 patients in Hungary). These numbers are similar to the estimation of Alzheimer Europe’s one (148,927 patients)^17^.

In previous Hungarian studies, the incidence of dementias was studied among residents of nursing homes. A study^18^ published in 1995 found the incidence of VaD to be more common compared to AD, while another study^19^ made no distinction in the etiology of dementia, but found that nearly half of the residents suffered from at least mild cognitive deficit.

In addition, in some of the studies^20–22^ small sample sizes (1500^20^ and 407^21^ participants) were used from randomly selected FM practices to extrapolate the prevalence of dementia in patients over 55 years old at population level. As a result, prevalence data were overestimated, leading to the number of patients with any type of dementia in Hungary in 2008 between 530 and 917,000.

We did not find Hungarian data about clinical criteria based VaD:AD ratio, but according to a neuropathology analysis, AD-type pathology was seen in 49.2% of VaD cases; pure AD pathology was found in 26.3% and pure VaD in 17.3% of the examined patients, which was close to the international results^23^.

In our study, the ratio of the VaD and AD diagnoses was reversed, 2.54 times more VaD diagnoses were assigned than AD (this ratio includes mixed diagnoses also). When accounting pure VaD and AD diagnoses (only 36,204 and 9165 patients over the six years, respectively), VaD:AD ratio increases to 3.95:1. Our results showed that both dementia and MCI were significantly underdiagnosed and the categorization of patients into dementia subtypes is also different from international data (Table 4).

**Table 4:** Estimation of prevalence of dementia and ratio of VaD and AD according to different sources.

Sources	Prevalence of dementia	Ratio of VaD:AD
International results	1600–1700^15,16^^a^ 1489^17^	1:3^10,11^
Previous Hungarian results	5300–9170^20–22^	1:1.52^23^
Our study	570	2.54:1

We summarized the prevalence of dementia from the literature. It is complicated to compare the results because the examined samples. The data shows number of patients per 100,000 inhabitants.

^a^Limited data are available from Hungary therefore we had to use estimations from the region.

In Hungary, the type of dementia is defined by neurological or psychiatric specialist services after family physician referral, so FM records were not included in our analysis. However, dementias are underdiagnosed worldwide in FM practices, the national underdetection rate is about 52% in the United Kingdom^24^ and roughly 75% in middle or lower income countries^25^, thereby fewer patients can be admitted to the specialist care. In addition, it is possible that patients with MCI and in early stages of dementia were managed in the FM practices and they were referred in later stages for specialty services, as suggested by the shorter survival time, the low number of MCI diagnoses preceding dementia and the high mortality of patients with dementia in our study.

The limited availability of AD biomarkers (CSF beta-amyloid and phospho-tau and amyloid PET imaging)^26^ might partially explain the low rate of AD diagnoses. The high percentage of patients without neuroimaging could also be a factor for this.

The survival of patients without neuroimaging was shorter by 1.24 years compared to patients with neuroimaging (data not shown) and the ratio of patients without neuroimaging was higher among patients diagnosed in psychiatric services alone. The shorter survival and the less frequent use of neuroimaging among patients diagnosed with psychiatric services alone might also be explained by the more severe stage of dementia with prominent behavioral and psychological symptoms at the time of referral.

In addition, the VaD:AD ratio was the lowest in patients diagnosed by neurological specialty services alone. It could be hypothesized that neurologists might more often notice the focal signs of cerebral circulatory problems during physical examination and indicate head imaging, as well as in the absence of symptomatic vascular lesions on imaging studies they less frequently diagnose VaD. This might also be supported by our result that AD patients more frequently received unspecific dementia diagnosis (mD) than VaD patients. Moreover, compared to Western countries (e.g. USA, UK, Germany), the incidence of stroke is 1.3–2 times higher in Hungary^27^, which may partly explain the higher incidence of VaD. Accurate differentiation between types of dementias is important not only for the choice of the ideal treatment, but also because of the quality of life of patients and their caregivers, which could be significantly different^28^.

There is a significant difference in the course of different types of dementia: survival is worse in VaD followed by all dementias and AD^29^. Patients with dementia have a higher incidence and risk of death from stroke. In addition, the presence of cardiovascular risk factors is higher in VaD than in any other forms of dementia^30^. The shortest survival seen in VaD might be explained by the higher risk of stroke and other cardiovascular diseases, the late complications of cerebral infarctions and by the finding that demented patients receive poorer quality of care with worse outcomes after stroke^31^. These observations are supported by the findings of Broulikova et al.^32^ from Czech registers on hospitalized dementia patients.

In our study, the median survival of patients with all dementias (3.01 years) was shorter than the published ones (3.2–6.6 years)^33^, being the shortest in VaD (2.25 years). All types of dementia have higher case fatality in men, similarly to the literature^29^.

The reasons for the difference are unclear, however, as mentioned above, late detection of dementia and diagnosis at a more advanced stage may contribute, as indicated by the very low proportion of MCI codes that precede dementia. In addition, differences in baseline cognitive performance and rate of decline across European regions might also contribute to the observed differences in survival^34^. In addition, palliative and supportive care for patients with dementia and their caregivers is limited, although improving in Hungary. Optimal collaborative care is necessary to properly treat the wide range of cognitive, emotional, social or physical complications associated with dementia^35^. Development of extensive cooperation can effectively improve quality of life and the survival, as well as reduce social and economic burden^36^.

## Limitations and strengths of the study

The NEUROHUN database allows us to estimate the number of patients with dementia and MCI at population level, with diagnoses confirmed by neurological and psychiatric providers, leading to more specific and reliable, but underestimated numbers of patients with dementia. Inclusion of data from FM practices could increase the number of identified patients, but without specifying the type of dementia. Dementia prevalence data are barely available from the Central and Eastern European regions and our results help to fill this gap and they are the first from a large sample size research from Hungary.

## Supplementary Information

Supplementary Information.

## Data Availability

Data sharing is not applicable to this article as no new data were created or analyzed in this study.
