# Supplementary material for: Dementia epidemiology in Hungary based on data from neurological and psychiatric specialty services
Source: Sci Rep. 2021 May 14;11:10333. doi: 10.1038/s41598-021-89179-3 (PMC8121883; doi:10.1038/s41598-021-89179-3)
Supplement: Supplementary file 1 — Supplementary Information. [file 41598_2021_89179_MOESM1_ESM.pdf]

DEMENTIA EPIDEMIOLOGY IN HUNGARY BASED ON DATA FROM NEUROLOGICAL AND PSYCHIATRIC SPECIALTY SERVICES

Nóra Balázs, András Ajtay, Ferenc Oberfrank, Dániel Bereczki, Tibor Kovács

Correspondence:

Tibor Kovács, e-mail: kovacs.tibor@med.semmelweis-univ.hu

Department of Neurology, Semmelweis University, Budapest, Hungary

H-1083 Budapest Balassa utca 6., Hungary

Supplementary information 1

The effect of gender and dementia subtype on age

Cell No.	Tukey HSD test; Approximate Probabilities for Post Hoc Tests Error: Between MS = 114,36, df = 2304E2 Error: Between MS = 114,36, df = 2304E2									
	**Gender**	**Dementia type**	**{1} (73,824)**	**{2} (74,879)**	**{3} (75,019)**	**{4} (70,157)**	**{5} (77,944)**	**{6} (76,558)**	**{7} (79,217)**	**{8} (75,245)**
**1**	Men	All dementias		*0,000035*	*0,000032*	*0,000032*	*0,000032*	*0,000032*	*0,000032*	*0,000032*
**2**	Men	AD	*0,000035*		0,998238	*0,000032*	*0,000032*	*0,000032*	*0,000032*	0,636752
**3**	Men	VaD	*0,000032*	0,998238		*0,000032*	*0,000032*	*0,000032*	*0,000032*	0,542726
**4**	Men	mD	*0,000032*	*0,000032*	*0,000032*		*0,000032*	*0,000032*	*0,000032*	*0,000032*
**5**	Women	All dementias	*0,000032*	*0,000032*	*0,000032*	*0,000032*		*0,000032*	*0,000032*	*0,000032*
**6**	Women	AD	*0,000032*	*0,000032*	*0,000032*	*0,000032*	*0,000032*		*0,000032*	*0,000032*
**7**	Women	VaD	*0,000032*	*0,000032*	*0,000032*	*0,000032*	*0,000032*	*0,000032*		*0,000032*
**8**	Women	mD	*0,000032*	0,636752	0,542726	*0,000032*	*0,000032*	*0,000032*	*0,000032*	

Two-way ANOVA, post hoc Tukey HSD matrix. The dependent variable was the age of the patients, the independents were the gender and the types of dementias. Significant differences were marked in italic
